# The Impact of Increased Heat on the Physical, Mental, and Social Health Domains of Adults in the United Arab Emirates in 2024

**DOI:** 10.7759/cureus.74951

**Published:** 2024-12-02

**Authors:** Alaa Al Hurini, Anas Nemmar, Karim Moawad, Rifa Khan, Jayakumary Muttappallymyalil

**Affiliations:** 1 College of Medicine, Gulf Medical University, Ajman, ARE; 2 Department of Community Medicine, College of Medicine, Gulf Medical University, Ajman, ARE

**Keywords:** climate change and its effect on life and health, exposure, health, heat exposure, mental, physical, social, uae

## Abstract

Objectives: The purpose of this research was to assess the impact of exposure to heat on the physical, social, and mental health domains of adults residing in the United Arab Emirates (UAE), where the region faces great increases in temperature due to climate change. Previous research has focused mainly on physical health outcomes; this research addressed the expansive impacts of mental and social health, which remain understudied in the region.

Methods: A cross-sectional study surveyed 397 adults in the UAE using a structured questionnaire. It captured all the factors on heat exposure duration, physical health symptoms, and self-reported measures of mental and social health while using the Depression, Anxiety, and Stress Scale (DASS) to assess mental health impact. The questionnaire was validated by experts in public health and psychology. Data analysis was done by using SPSS version 28 (IBM Corp., Armonk, NY). A chi-square test was performed to determine the association between heat exposure and health outcomes. A p-value ≤ 0.05 was considered statistically significant.

Results: The study of 397 adults in the UAE found that 331 (83.4%) are exposed to heat for more than two hours per day and 66 (16.6%) for two hours or less. The most commonly reported health problem was heat exhaustion (n = 343 (86.4%)). Other common symptoms include heat rash (n = 81 (20.4%)), heat cramps (n = 81 (20.4%)), heat syncope (n = 163 (41.1%)), and heat stroke (n = 171 (43.1%)). Additionally, the study found that 179 (45.1%) respondents reported suffering from anxiety, 141 (35.5%) respondents reported suffering from depression, and stress prevalence was 11.6% for 46 participants. The study also found that a significant minority reported social isolation (n = 79 (19.9%)), loneliness (n = 80 (20.2%)), lack of social connectedness (n = 70 (19.9%%)), low quantity or quality of contact with others (n = 100 (25.2%)), lack of feeling of belonging (n = 96 (24.2%)), and lack of fulfilling relationships (n = 87 (21.9%)).

Conclusion: Rising heat exposure in the UAE is shown to be associated with adverse effects across physical, mental, and social health domains. These findings emphasize the need for public health interventions that address not only the physical dangers of rising heat but also its psychological and social impacts. Policymakers and healthcare providers should consider comprehensive strategies to mitigate these multifaceted health risks in the face of rising temperatures.

## Introduction

The impact of climate change on human health has become a critical concern in recent times. As temperatures continue to rise, the threat of extreme heat and its consequences on human health is becoming more apparent. Exposure to high temperatures can result in a range of physical health problems, including dehydration, heat-related illnesses, cardiovascular damage, and even renal failure [1-4]. Moreover, exposure to extreme heat can also have a significant impact on mental health, leading to increased anxiety and other psychological symptoms [5,6]. Vulnerable populations, including the elderly and individuals with disabilities, are disproportionately affected by these extreme conditions [7-9]. In the Eastern Mediterranean, the impact of the hot and dry summers on physical and emotional well-being is also a cause for concern, with poor management of waste and uncontrolled carbon dioxide emissions being responsible for an increase in cancer rates and cardiovascular diseases [10-13]. While global studies have identified the health risks of heat, there is a scarcity of research focusing on the specific effects of extreme heat on physical, mental, and social health in the United Arab Emirates (UAE). This study aims to examine the impact of extreme heat and its consequences on different domains of human health. The study will shed light on the various ways in which extreme heat can affect human health, both physically and emotionally, and provide valuable insights into the urgent need for proactive measures to mitigate the effects of climate change on human health.

## Materials and methods

The study design adopted for this research was cross-sectional, aimed at fulfilling the outlined objectives efficiently. The study population comprised adults residing in Ajman, UAE, with inclusion criteria set for individuals above 18 years of age of any nationality or sex, capable of providing consent. Exclusion criteria were defined to exclude unwilling participants or those refusing consent, ensuring data relevance. The initial sample size of 369 adults was determined using the Z^2(PQ)/L^2 formula, with adjustments made for a 10% non-response rate, resulting in a final sample size of 409. Data collection was conducted from October 2022 to December 2022. After excluding responses that did not fulfill the inclusion criteria or had incomplete responses, the final sample size that was analyzed was 397.

Convenience sampling was employed to recruit participants from various settings in Ajman, including labor camps, Thumbay University Hospital, and Gulf Medical University. The structured questionnaire captured all the factors on heat exposure duration, physical health symptoms of heat illness, and self-reported measures of mental and social health while using the Depression, Anxiety, and Stress Scale-21 Items (DASS-21) to assess mental health impact. The questionnaire was validated by experts in public health and psychology, and a pilot study with five adults was conducted to assess feasibility and comprehension. Data collection was conducted with institutional review board (IRB) approval, ensuring privacy, confidentiality, and voluntary participation. The research began in January 2022 and was completed in February 2023.

Ethical considerations were paramount, with approval obtained from the Gulf Medical University Institutional Review Board with reference number IRB/COM/STD/71/April-2022 and informed consent collected from participants. No personal information was collected to maintain anonymity and confidentiality. Data analysis involved descriptive and inferential statistics using SPSS version 28 (IBM Corp., Armonk, NY), with results expressed in frequencies and percentages. Chi-square tests were performed to determine associations between variables, with statistical significance set at p ≤ 0.05. Overall, rigorous ethical standards were adhered to throughout the study, ensuring the protection of human subjects and the integrity of the research findings.

## Results

In this study, regarding the sociodemographic profile of the participants, most were men (Table 1), reflecting the gender composition commonly found in labor camps in Ajman. The majority of these workers hailed from Asian countries, specifically Pakistan, India, and Bangladesh, indicating a significant representation of South Asian expatriates in the labor force within the UAE, particularly in outdoor occupations. Given the setting of a labor camp, the study naturally skewed toward individuals engaged in manual labor, often outdoors, under the harsh climatic conditions of Ajman. The results indicate that gender, nationality, employment status, and marital status are significantly associated with the duration of heat exposure. Women and unemployed participants were found to have shorter heat exposure periods, while those from Southeast Asia had longer exposure than participants from other regions. Additionally, single participants had longer heat exposure periods compared to their married counterparts. This demographic is critical for assessing the impact of heat exposure, as outdoor workers are particularly vulnerable to heat-related health issues due to prolonged periods spent in high-temperature environments. The focus on labor camps also implies that the study addressed a group that might experience unique physical, social, and mental health challenges. The combination of strenuous physical activity, high heat exposure, and potentially limited access to healthcare or social support systems places this population at an increased risk of health problems associated with heat. This specific sociodemographic focus allows for a targeted examination of the impact of heat on a group that is both highly exposed and potentially more susceptible to the adverse health effects of high temperatures.

**Table 1:** Association Between Sociodemographic Characteristics and Duration of Daily Heat Exposure Among Adults in Ajman, UAE (2024) The chi-square test was used for statistical analysis.

Sociodemographic characteristics	Groups	Duration of heat exposure		p-value
		Less than or equal to 2 hours	Greater than 2 hours	
		Number (%)	Number (%)	
Age group in years	Less than 30 years	34 (19.2)	143 (80.8)	0.14
	Greater than or equal to 30 years	32 (14.5)	188 (85.5)	
Gender	Male	48 (12.9)	325 (87.1)	0.001
	Female	18 (75)	6 (25)	
Nationality	Southeast Asian region	16 (8.6)	171 (91.4)	<0.001
	Eastern Mediterranean region	42 (21.2)	156 (78.8)	
	Others	8 (66.7)	4 (33.3)	
Education level	Up to middle school	24 (25.3)	71 (74.7)	0.06
	High school and above	41 (17.4)	200 (82.6)	
Employment status	Employed	52 (14)	320 (86)	<0.001
	Unemployed	14 (73.7)	5 (26.3)	
Marital status	Single	30 (30)	70 (70)	<0.001
	Married	36 (12.1)	261 (87.9)	

The results indicate that the duration of work, hours worked in a day, and the type of occupation have a statistically significant association with the duration of heat exposure (Table 2). Specifically, participants who have been working for more than five years and those who work for more than eight hours a day are more likely to be exposed to heat for a longer duration.

**Table 2:** Association Between Details of Occupation and Duration of Daily Heat Exposure Among Adults in Ajman, UAE (2024) The chi-square test was used for statistical analysis.

Details of occupation	Groups	Duration of heat exposure		p-value
		Less than or equal to 2 hours	Greater than 2 hours	
		Number (%)	Number (%)	
Duration of work in years	Less than 5 years	21 (16)	110 (84)	<0.001
	5-9 years	31 (27.4)	82 (72.6)	
	Greater than or equal to 10 years	14 (9.2)	139 (90.8)	
Break in between work	Yes	59 (16)	309 (84)	0.16
	No	7 (25)	21 (75)	
Hours worked in a day	Less than or equal to 8 hours	24 (9.6)	225 (90.4)	<0.001
	8-10 hours	32 (34)	62 (66)	
	Greater than 10 hours	10 (18.9)	43 (81.1)	

The results indicate that the presence of heat exhaustion and heat stroke are statistically significant factors associated with the duration of heat exposure (Table 3), with p-values of 0.032 and <0.001, respectively. This suggests that participants who were exposed to heat for more than two hours were more likely to experience heat exhaustion and heat stroke than those who were exposed for less than or equal to two hours.

**Table 3:** Association Between Physical Health and Duration of Daily Heat Exposure Among Adults in Ajman, UAE (2024) The chi-square test was used for statistical analysis.

Physical health issues	Groups	Duration of heat exposure		p-value
		Less than or equal to 2 hours	Greater than 2 hours	
		Number (%)	Number (%)	
Heat rashes	Present	15 (18.5)	66 (81.5)	0.3
	Absent	49 (15.7)	264 (84.3)	
Heat cramps	Present	13 (16)	68 (84)	0.5
	Absent	53 (16.8)	263 (83.2)	
Heat edema	Present	6 (12.8)	41 (87.2)	0.3
	Absent	60 (17.1)	290 (82.9)	
Heat exhaustion	Present	62 (18.1)	281 (81.9)	0.032
	Absent	4 (7.4)	50 (92.6)	
Heat syncope	Present	21 (12.9)	142 (87.1)	0.06
	Absent	45 (19.2)	189 (80.8)	
Heat stroke	Present	42 (24.6)	129 (75.4)	<0.001
	Absent	23 (11.1)	185 (88.9)	

Notably, despite the physical stress of prolonged heat exposure, we found no significant association between heat exposure duration and anxiety or depression scores (p>0.05). This could suggest that the direct impact of heat exposure on mental health outcomes might be more complex or influenced by additional factors, such as social support or occupational roles. However, there was a statistically significant association between stress and duration of heat exposure (p = 0.009) (Table 4).

**Table 4:** Association Between Mental Health and Duration of Daily Heat Exposure Among Adults in Ajman, UAE (2024) The chi-square test was used for statistical analysis.

Details of occupation	Groups	Duration of heat exposure		p-value
		Less than or equal to 2 hours	Greater than 2 hours	
		Number (%)	Number (%)	
Depression	Present	27 (19.1)	114 (80.9)	0.18
	Absent	39 (15.2)	218 (84.8)	
Anxiety	Present	27 (15.1)	152 (84.9)	0.28
	Absent	39 (17.7)	181(82.3)	
Stress	Present	14 (30.4)	32 (69.6)	0.009
	Absent	52 (14.7)	301 (85.3)	

Results show a significant association between the social health issue of being disconnected from resources of health protection and promotion and the duration of heat exposure (Table 5) (p = 0.007). There is also a significant association between the social health issue of lack of feeling of belonging and the duration of heat exposure (p = 0.007), as well as the social health issue of lack of fulfilling relationships and the duration of heat exposure (p = 0.02).

**Table 5:** Association Between Social Health Issues and Duration of Daily Heat Exposure Among Adults in Ajman, UAE (2024) The chi-square test was used for statistical analysis.

Social health issues	Groups	Duration of heat exposure		p-value
		Less than or equal to 2 hours	Greater than 2 hours	
		Number (%)	Number (%)	
Social isolation	Yes	11 (16.7)	68 (20.5)	0.30
	No	55 (83.3)	263 (79.5)	
Disconnected from social networks	Yes	12 (18.2)	83 (25.1)	0.15
	No	54 (81.8)	248 (74.9)	
Disconnected from resources of health protection and promotion	Yes	9 (13.6)	94 (28.4)	0.007
	No	57 (86.4)	237 (71.6)	
Loneliness	Yes	10 (15.2)	70 (21.1)	0.17
	No	56 (84.8)	261 (78.9)	
Lack of social connectedness	Yes	11 (16.7)	68 (20.5)	0.30
	No	55 (83.3)	263 (79.5)	
Low quality or quantity of contact with others	Yes	15 (22.7)	85 (25.7)	0.37
	No	51 (77.3)	246 (74.3)	
Lack of feeling of belonging	Yes	8 (12.1)	88 (26.6)	0.007
	No	58 (87.9)	243 (73.4)	
Lack of fulfilling relationships	Yes	8 (12.1)	79 (23.9)	0.02
	No	58 (87.9)	252 (76.1)	
Lack of engagement with others	Yes	12 (18.2)	79 (23.9)	0.2
	No	54 (81.8)	252 (76.1)	

## Discussion

The study received a 95% response rate with 397 participants with complete responses. The research revealed a predominantly male demographic, consistent with the gender composition typically found in labor camps in the region, with a significant representation of South Asian expatriates, particularly from Pakistan, India, and Bangladesh. This demographic specificity is crucial for understanding the impact of heat exposure, given the nature of outdoor manual labor prevalent in such settings. The study's focus on labor camp inhabitants allows for a targeted examination of the intersection between heat exposure and health outcomes, considering the unique challenges faced by this population, including limited access to healthcare and social support systems.

Moreover, the study highlights the significant association between occupational factors such as duration of work, hours worked per day, and type of occupation with the duration of heat exposure. The findings align with existing literature indicating that prolonged exposure to high temperatures exacerbates health risks, particularly among vulnerable groups such as the elderly, children, and individuals with pre-existing medical conditions [14-17]. The focus on labor camps also implies that the study addressed a group that might experience unique physical, social, and mental health challenges as previous research concluded that occupational factors such as labor workers and farmers were affected the most by exposure to heat and had many symptoms related to heat illness [18,19]. The combination of strenuous physical activity, high heat exposure, and potentially limited access to healthcare or social support systems places this population at an increased risk of health problems associated with heat, and as other studies have found loss of productivity as a consequence of heat exposure [20], it is important to address the impacts of heat exposure to provide better coping methods.

Furthermore, this study identifies specific heat-related ailments such as exhaustion and stroke that are significantly associated with prolonged exposure, emphasizing the critical importance of addressing occupational health hazards in high-temperature environments. They highlight the importance of taking steps to protect against the negative effects of heat and address the root causes of climate change in order to reduce the risks to human health. In other literature in the Middle East with similar climates as the UAE, there have been positive correlations between morbidity and mortality and heat exposure [21,22]; some even found that there is a pattern between cardiovascular failure and excessive heat [23], relating it to dehydration.

This study found that the majority of the individuals were exposed to heat for more than two hours per day, and most individuals reported experiencing symptoms of heat-related illness. A significant proportion of the individuals also reported stress and a lesser degree of depression and anxiety due to heat exposure. The prevalence of heat-related illnesses and the impact on mental, physical, and social health in the UAE varied. In a Malaysian study conducted in 2018, the urban heat island effect was cited as a significant factor [24]. This effect causes the heat to be absorbed and retained in the country, unlike in the UAE. The prevalence is shown in Table 6.

**Table 6:** Malaysian Study in Comparison to Our UAE Study

Symptoms	Malaysian study [24] (N = 558)	Our UAE study (N = 397)
Heat exhaustion	89.4%	86.4%
Heat stroke	86.7%	43.1%
Heat cramps	81.9%	20.4%
Anxiety	79.0%	45.1%
Depression	74.4%	35.5%
Reduced outdoor activity	90.0%	19.9%

Compared to this Malaysian study (Table 6), all heat-related illnesses are much more prevalent, as well as mental and social health effects. This difference may reflect the UAE's infrastructure and regulatory efforts to protect workers in increased heat environments. Also, it has been concluded that the majority of Ajman's laborers in this study have been working for 10 or more years and had rest breaks; this suggests that they have more experience in combating heat effects, which is similar to another study findings suggesting people who experience the detrimental effects of heat on their health tend to adopt more of the behavioral tendencies measured by the adaptation index than those perceiving little or none [25]. Furthermore, most of the participants in our study reported at least one coping mechanism and lived in labor camps. In the Malaysian study, there is a significant factor that also played a great role, that is, the urban heat island effect. This effect causes the heat to be absorbed and retained in the country, unlike in the UAE.

The mental health impacts observed in our study (Table 4) align with global findings indicating that extreme heat is linked to increased hospital admissions for conditions such as anxiety and depression, with a study indicating an increase of 8% more admissions for mental health on days with increased heat in comparison to days without increased heat [26]. Another study conducted in Northern Vietnam measured the relationship between heat waves and hospital admissions for mental disorders. It concluded that heat waves increased the risk for admission in the whole group of mental disorders for more persistent heat waves of at least three days when compared with non-heat wave periods [27]. This is very important because many studies have shown the impact of heat on mental health, and some studies even found that psychological distress was more common than usual in relation to heat [28]. These results of the impact of heat on mental health are parallel with the results we received in our studies, although the methodology and analysis were different.

Beyond physical health effects, this research has found that there was a significant association between increased duration of heat exposure and social health issues such as feeling disconnected from resources of health protection and promotion, having a lack of feeling of belonging, and a lack of fulfilling relationships (Table 5). Recent research suggests that there is an association between heat and its impact on social health, as it prevents people from socializing directly and indirectly [29].

The study highlights the negative impact of heat exposure on the physical and mental health and social well-being of individuals in the emirate of Ajman population. It is crucial for individuals to take steps to protect themselves from the negative effects of heat, such as seeking treatment when necessary, trying to reduce exposure to heat, and utilizing coping mechanisms to manage the effects of heat. Moreover, it is essential for the government and policymakers to take action to mitigate the negative effects of heat on the population, such as improving infrastructure and providing resources for health protection and promotion to improve productivity and working conditions. Significant efforts are already underway in the UAE to address these issues. However, given the extreme heat in the region, these measures can be further enhanced. In some regions, 30%-40% of the yearly daylight hours will be too hot for employment. The social and economic consequences will be significant, with global gross domestic product (GDP) losses of more than 20% by 2100 if climate change continues on its current path [30]. This further stresses the urgent need to combat the rising heat levels.

This study has limitations that should be addressed. The sample specificity on labor camps in Ajman targets a predominantly South Asian male demographic, which limits generalizability to other populations such as women, children, or people of different ethnic backgrounds in the UAE. Since the study adopted a cross-sectional design, it provided a snapshot of rising heat impact; it lacks longitudinal data to assess long-term impacts on physical, mental, and social health. In addition, self-reported data on health symptoms and coping mechanisms may introduce bias, as participants might underreport or overreport symptoms based on personal perceptions. Lastly, the study might benefit from a more in-depth focus on mental health metrics beyond the DASS-21 to better understand the specific psychological effects of heat exposure.

Recommendations

To combat the health risks associated with increased heat exposure, we propose the following recommendations. For individuals, take precautions during periods of extreme heat by staying hydrated and seeking air conditioning or shade. For employers, implement extended rest periods, ensure accessible hydration points, and provide heat-resistant protective gear to minimize physical strain from prolonged outdoor work. For policymakers, develop and enforce heat-health action plans with labor camp-specific measures, including cooling installations, social support resources, and health screening for early detection of heat-related illnesses. As global recommendations, nations should work together to raise awareness and identify causes to reverse changes. Effects should focus on urban greening initiatives, efficient warning systems, and comprehensive climate policies to alleviate the socioeconomic and health burdens of increased heat.

## Conclusions

This study illustrates a clear association between heat exposure and adverse health effects among adults in Ajman, UAE, where 83.4% of individuals were exposed to heat for more than two hours daily and 86.8% experienced heat exhaustion. Importantly, our findings showed high levels of psychological distress and social isolation among participants, indicating that heat exposure negatively affects physical, mental, and social health.

## Appendices

Questionnaire

Sociodemographic Characteristics

Age: ………… Gender: M/F    Nationality: ............. Marital status:  M /S  Level of education: ....................

Living status: With family …  Without family …          Weight: ……….. Height: ……….

Occupation Details

Table 7 shows the questionnaire on the occupation details of the participants.

**Table 7:** Occupation Details

What is your occupation?	
For how many years have you been working?	
Do you get a rest break in between your work?	Yes/no
How many hours do you work in a day?	
Duration of heat exposure during the day	
Duration of heat exposure during work hours?	

Physical Health Domain

The participants were asked if they had ever experienced heat rashes, heat cramps, heat edema, heat exhaustion, heat syncope, or heat stroke when exposed to a hot, humid environment (Table 8).

**Table 8:** Impact of Heat on Physical Health

Physical conditions	Symptoms	Yes	No
Heat rashes	Tiny red spots usually appear on the neck, upper chest, groin, and in elbow creases		
	Severe itching		
Heat cramps	Sharp pains in the muscles		
Heat edema	Swelling noticeable in the ankles		
Heat exhaustion	Headache		
	Nausea		
	Dizziness		
	Weakness		
	Irritability		
	Thirst		
	Heavy sweating		
	Elevated body temperature		
	Decreased urine output		
	Muscle cramps		
	Breathlessness		
	Palpitations		
	Skin pale, cool and moist		
	Visual disturbances		
Heat syncope	Dizziness		
	Fainting		
	Lightheadedness from standing too long		
Heat stroke	Confusion		
	Altered mental status		
	Slurred speech		
	Loss of consciousness (coma), partial		
	Loss of consciousness (coma), complete		
	Hot, dry skin		
	Seizures		
	Very high body temperature		

Mental Health Domain

Following the Depression, Anxiety, and Stress Scale-21 Items (DASS-21), the participants were asked to select what applies to them during frequent heat exposure (Table 9).

**Table 9:** DASS-21 to Assess the Impact on Mental Health DASS-21: Depression, Anxiety, and Stress Scale-21 Items

No.	Questions	Did not apply to me at all (never)	Applied to me to some degree or some of the time (sometimes)	Applied to me to a considerable degree or a good part of the time (often)	Applied to me very much, or most of the time (almost always)
1	I found it hard to wind down.				
2	I was aware of the dryness of my mouth.				
3	I couldn't seem to experience any positive feelings at all.				
4	I experienced breathing difficulty.				
5	I found it difficult to work up the initiative to do things.				
6	I tended to overreact to situations.				
7	I experienced trembling.				
8	I felt that I was using a lot of nervous energy.				
9	I was worried about situations in which I might panic and make a fool of myself.				
10	I felt that I had nothing to look forward to.				
11	I found myself getting agitated.				
12	I found it difficult to relax.				
13	I felt downhearted and blue.				
14	I was intolerant of anything that kept me from getting on with what I was doing.				
15	I felt I was close to panic.				
16	I was unable to become enthusiastic about anything.				
17	I felt that I was rather touchy.				
18	I felt that life was meaningless.				
19	I felt scared without any good reason.				
20	I was aware of the action of my heart in the absence of physical exertion.				
21	I felt I wasn't worth much as a person.				

Social Health Domain

The participants were asked which of the following social health issues shown in Table 10 they experienced in a hot/humid environment.

**Table 10:** Social Health Domain

Social health issues	Yes	No
Social isolation		
Disconnected from social networks and supports		
Disconnected resources for health protection and health promotion		
Loneliness		
Lack of social connectedness		
Low quantity or quality of contact with others		
Lack of feeling of belonging		
Lack of fulfilling relationships		
Lack of engagement with others		

Coping Strategies

Table 11 shows the questionnaire for the coping strategies the participants used/preferred.

**Table 11:** Coping Strategies

Which of the following do you use/prefer?	Yes	No
Visit a clinic for treatment		
Work in a cooler, less humid environment, if possible		
Sit or lie down in a cool place		
Remove outer clothing including shoes and socks		
Cool the body		
Drink water, clear juice		
Cold water or ice bath		
Wet the skin		
Place cold wet clothes on the skin		
Place cold wet clothes or ice on the head, neck, armpits, and groin, or soak the clothing with cool water		
Circulate air around to speed cooling		
Participate in social activity in indoor places		
